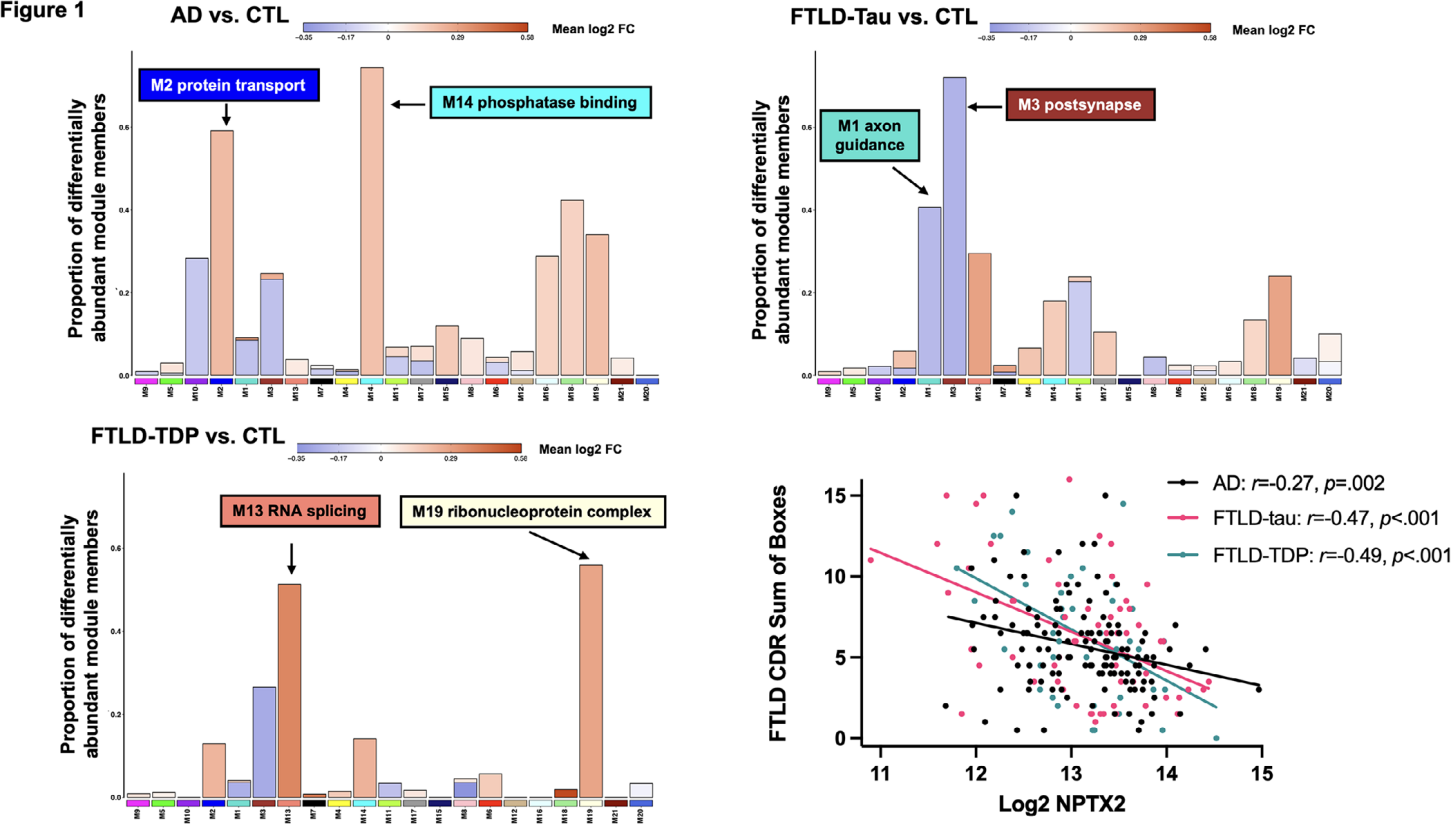# Large‐scale cerebrospinal fluid proteomics identifies molecular signatures of disease progression across Alzheimer's disease and sporadic frontotemporal lobar degeneration

**DOI:** 10.1002/alz70856_107629

**Published:** 2026-01-11

**Authors:** Rowan Saloner, Joshua Downer, Julia D Webb, Argentina Lario Lago, Peter A. Ljubenkov, Lawren VandeVrede, Adam M. Staffaroni, Emily W. Paolillo, Salvatore Spina, Lea T. Grinberg, Renaud La Joie, Gil D. Rabinovici, Joel H Kramer, Maria Luisa Gorno Tempini, Bruce L. Miller, Howard J. Rosen, Julio C. Rojas, Jennifer S. Yokoyama, William W. Seeley, Adam L. Boxer, Kaitlin B Casaletto

**Affiliations:** ^1^ University of California San Francisco, San Francisco, CA, USA; ^2^ University of California, San Francisco, San Francisco, CA, USA; ^3^ Memory and Aging Center, UCSF Weill Institute for Neurosciences, University of California, San Francisco, San Francisco, CA, USA; ^4^ Memory and Aging Center, UCSF Weill Institute for Neurosciences, University of California San Francisco, San Francisco, CA, USA; ^5^ Memory and Aging Center, Weill Institute for Neurosciences, University of California San Francisco, San Francisco, CA, USA; ^6^ Memory and Aging Center, Weill Institute for Neurosciences, University of California, San Francisco, San Francisco, CA, USA

## Abstract

**Background:**

Alzheimer's disease (AD), frontotemporal lobar degeneration (FTLD), and other neurodegenerative diseases are characterized by pathological protein misfolding, yet postmortem neuropathological measures collectively explain less than half the variance in antemortem clinical progression. We leveraged large‐scale cerebrospinal fluid (CSF) proteomics to identify overlapping and distinct molecular signatures that help explain clinical severity across patients with AD, FTLD‐tau, and FTLD‐TDP.

**Method:**

CSF was assayed via aptamer‐based proteomics (SomaScan v4.1 >7k proteins) in 132 symptomatic AD patients (positive CSF *p*‐Tau181/Ab_42_ ratios and/or amyloid PET), 64 sporadic FTLD‐tau patients, 42 sporadic FTLD‐TDP patients, and 72 AD biomarker‐negative controls. FTLD cases screened negative for autosomal dominant FTLD mutations and were classified via either autopsy confirmation (65% of cases; FTLD‐tau: progressive supranuclear palsy [PSP], corticobasal degeneration, Pick's; FTLD‐TDP: types A‐C) or clinical diagnosis with high etiologic specificity (35% of cases; FTLD‐tau: PSP‐RS; FTLD‐TDP: semantic variant primary progressive aphasia). Clinical severity was measured via CDR®+NACC FTLD sum of boxes. Differential abundance and co‐expression network analyses modeled proteomic differences across groups and in relation to clinical severity.

**Result:**

We identified 1,026 differentially abundant proteins (DAPs) across diseases (omnibus ANOVA FDR‐*p* <.05) that mapped onto 21 co‐expression modules. Most modules harbored DAPs shared by >1 disease vs. control, suggesting common molecular signatures across diseases. Protein degradation modules exhibited overrepresentation of DAPs that were elevated in AD (module M14 phosphatase binding: 74% DAPs, M2 protein transport: 59% DAPs), neuronal modules had overrepresentation of DAPs that were reduced in FTLD‐tau (M3 postsynapse: 73% DAPs, M1 axon guidance: 40% DAPs), and RNA metabolism modules had overrepresentation of DAPs that were reduced in FTLD‐TDP (M19 ribonucleoprotein complex: 56% DAPs, M13 RNA splicing: 51% DAPs). Neuronal modules harbored the largest proportion of proteins negatively correlated with clinical severity across the full sample (M3: 79%, M1: 59%; *p*s<.05), including targets that outperformed CSF neurofilament light in predicting clinical severity across all three disease groups (e.g., NPTX2, IRF1, TMEM132B).

**Conclusion:**

Findings highlight overlapping CSF proteomic alterations across AD and sporadic FTLD. Neuron‐enriched protein communities robustly tracked with clinical severity across diseases, underscoring their potential as transdiagnostic biomarkers of disease progression and therapeutic targets for all aging brains.